# Pressure Protein Denaturation Compared to Thermal and Chemical Unfolding: Analyses with Cooperative Models

**DOI:** 10.1021/acs.jpcb.4c07703

**Published:** 2025-01-17

**Authors:** Joachim Seelig, Anna Seelig

**Affiliations:** Biozentrum, University of Basel, Spitalstrasse 41, CH-4056 Basel, Switzerland

## Abstract

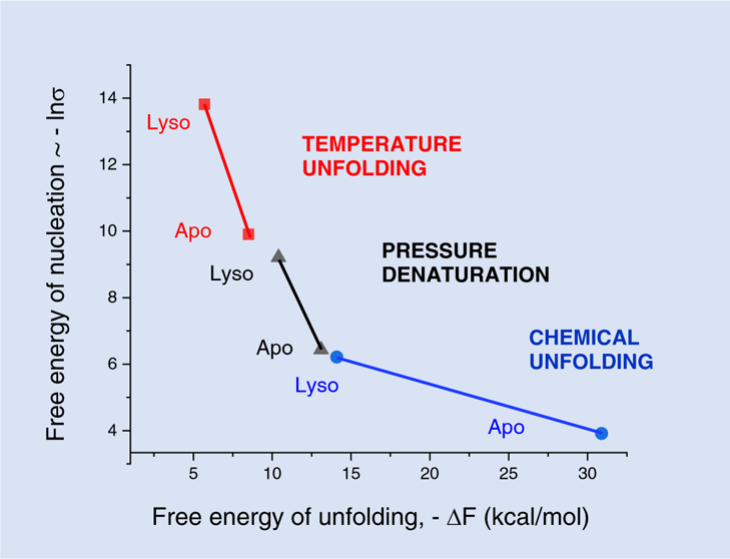

The thermodynamics of pressure-induced protein denaturation could so far not be directly compared with protein denaturation induced by temperature or chemical agents. Here, we provide a new cooperative model for pressure-induced protein denaturation that allows the quantitative comparison of all three denaturing processes based on their free energy, enthalpy, entropy, and cooperativity. As model proteins, we use apolipoprotein A-1 and lysozyme. The comparison shows that heat-induced unfolding is the most cooperative process. It is characterized by large positive enthalpies and entropies and (due to enthalpy–entropy compensation) small negative free energies. Pressure denaturation is less cooperative. The entropies and enthalpies are less positive, and the resulting free energies are more negative. Chemically induced unfolding is the least cooperative and shows the most negative free energies, in particular, if guanidinium hydrochloride (exhibiting a high binding affinity to certain proteins) is used as a denaturant. The three unfolding processes differ not only with respect to their cooperativity and the thermodynamic parameters but also with respect to the volume changes, suggesting structural differences of the denatured proteins. Using cooperative models thus yields significant new insights into the protein unfolding/folding processes.

## Introduction

Proteins can be inactivated or unfolded by heating,^1−5^ chemical agents,^6−8^ or high pressure.^9,10^ Unfolding has been monitored by various spectroscoptic techniques that reveal structural changes. Quantitative thermodynamic information in terms of the heat capacity change, *c*_p_(*T*), has been gained by differential scanning calorimetry (DSC). We demonstrated recently, that numerical integration of the heat capacity profile *c*_p_(*T*) yields the temperature profiles of enthalpy, Δ*H*(*T*), entropy, Δ*S*(*T*), and free energy, Δ*G*(*T*), without resorting to a model.^11^ With this new experimental approach it became possible to test different unfolding models.

A cooperative model for protein denaturation was proposed by Zimm–Bragg^12^ in 1959, but was widely ignored. During the last 10 years, we have shown that the Zimm–Bragg theory can be successfully applied to protein denaturation^13−17^ and presented comprehensive statistical-mechanical thermodynamic models for thermal^11,18,19^ and chemical^20−22^ unfolding. The experimentally obtained sigmoidal temperature profiles for enthalpy and entropy and the trapezoidal temperature profile for the free energy can be fitted with these new cooperative models, but not with the chemical two-state model used for more than 60 years.^11^

Here we extend our cooperative model to the analysis of protein denaturation under high pressure. We first compare the conventional chemical two-state model with a statistical two-state model. The focus of this work is however on a cooperative model of protein unfolding following, our previous proposals on thermal and chemical protein unfolding.^11,18,19,22^ With these models based on the same principles, it becomes possible to qunatitatively compare pressure denaturation with thermal and chemical denaturation.

## Theory

The pressure dependence of protein stability is usually monitored by following a spectroscopic intensity *I*(*p*). The intensity change upon pressure application can be used to calculate an equilibrium constant *K*_NU_(*p*)^9^
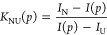
1where *I*_N_ and *I*_U_ are spectroscopic intensities of the native and the unfolded (denatured) protein. With this definition, the native protein has a small equilibrium constant. The fraction of unfolded protein is given by
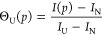
2Three caveats should be noted. First, the spectroscopic intensity *I*(*p*) is only an indirect measure of protein unfolding, that is, it may correlate only approximately with changes in protein stability, protein function, or protein volume. Second, the intensity *I*_U_ may not represent the fully unfolded protein. The problem of incomplete protein unfolding under most experimental conditions has been discussed by Sorokina.^23^ Third, proteins are compressible even at low pressure. An example is Staphylococcal nuclease (SNase) (cf. Figure 5 in ref (24)). The compressibility term κ = (1/*V*)(∂*V*∂*p*) is not considered in the following. It is not reflected in the spectroscopic measurements (cf. Figure 4 in ref (25)).

### Chemical Equilibrium Two-State Unfolding

In this model, pressure-unfolding is assumed to follow a two-state chemical equilibrium between a native N and an unfolded U protein. The pressure dependence of the chemical equilibrium constant is given by^9^
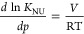
3aFor the chemical equilibrium two-state model, a constant volume reduction Δ*V*_0_ is assumed. Proteins are rather incompressible and Δ*V*_0_ is the decrease in protein volume upon applying pressure. Together with the pressure *p*_0_ at the midpoint of unfolding, Δ*V*_0_ is the second fit parameter to simulate the unfolding transition leading to
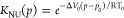
3bΔ*V*_0_ is negative and usually only 1–5% of the total protein volume.

The pressure dependence of the Gibbs free energy is
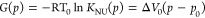
4*G*(*p*) is a linear function of pressure. At *p* = *p*_0_ the equilibrium constant is *K*_NU_(*p*_0_) = 1. The native and the unfolded protein have the same concentration, and the free energy *G*(*p*_0_) = 0. For *p* < *p*_0_ and a negative Δ*V*_0_, the free energy becomes positive. The native protein at low pressure has a large positive free energy, which is against common expectations of a stable native protein.

The fraction of unfolded protein is
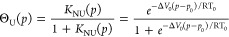
5

### Statistical–Mechanical Thermodynamic Model of Two-State Unfolding

In this model, the protein flips between two energy states. The native protein is the reference state with energy *E*_N_ = 0. The unfolded state has the energy *E*_U_(p) = Δ*V*_0_(*p* – *p*_0_). The partition function of this two-state system is (for details see refs (11,19,21))
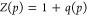
6
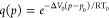
7The fraction of unfolded protein is
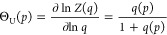
8This equation is analogous to eq 5 of the chemical equilibrium model.^22^ However, there is a significant difference between the two models with respect to the pressure dependence of the free energy. The free energy of the statistical-mechanical model is
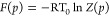
9As long as the N-state is predominant, the free energy of the native state is close to zero. For *p* = *p*_0_, the free energy is not zero as in the chemical equilibrium model, but is *F*(*p*_0_) = −RT_0_ ln 2. For *p* > *p*_0_, the free energy *F*(*p*) coincides with *G*(*p*) of the chemical equilibrium two-state model.

### Cooperative Multistate Unfolding Model

The cooperative multistate model assumes the interaction of ν molecular elements (e.g., amino acid residues or potein subdomains). Each element undergoes a transition from a native state “*n*” to a denatured state “*u*”. This could also be a small shift between subdomains reducing the total volume. The model has been applied previously to thermal and chemical protein unfolding.^11,18,19,22^ In the present case of pressure unfolding the *n* → *u* transition is associated with a small volume change Δ*v*_0_ per amino acid residue or subdomain. Δ*v*_0_ is an average value over all cooperative elements. Each element is assumed to flip between a native state of energy 0 and an unfolded state of energy Δ*v*_0_(*p* – *p*_0_). The cooperative interaction is characterized by a cooperativity parameter σ, which is also an average value. The partition function of the system is
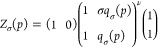
10
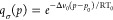
11The fraction of unfolded protein is
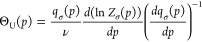
12Finally, the pressure dependence of the free energy is
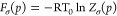
13The fit parameters in the simulation of the thermodynamic properties are thus the cooperativity parameter σ, the volume change Δ*v*_0_ per cooperative element and the number *ν* of cooperative elements participating in denaturation.

**Figure 1:**
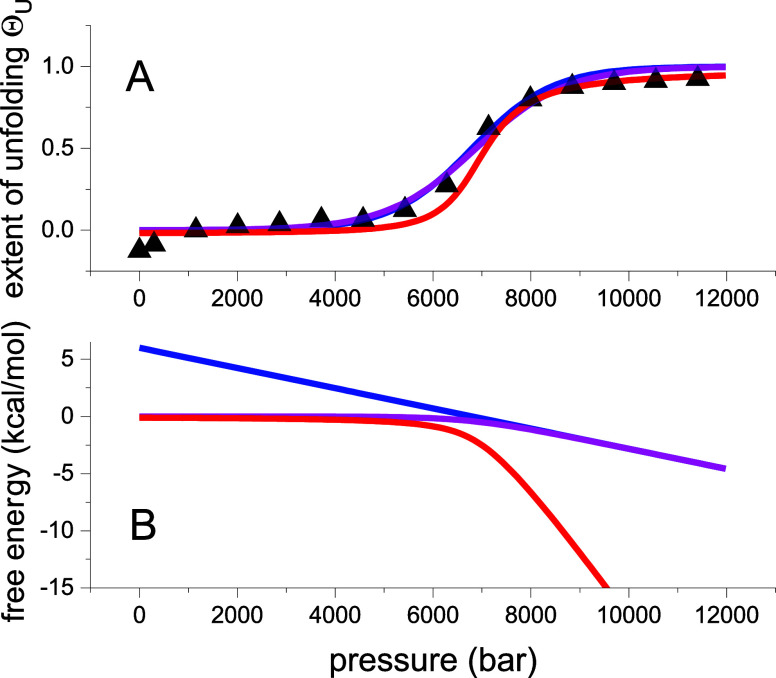
Comparison of three unfolding models. (A) (Δ) Experimental data of pressure-induced unfolding of lysozyme^26^ (details in Figure 2). Extent of unfolding Θ_U_(*p*). The solid lines represent the best fit of each model to the experimental lysozyme data. Blue line: chemical two-state model with Δ*V*_0_ = −37 mL/mol and *p*_0_ = 6800 bar. Magenta line: statistical-mechanical two-state model with Δ*V*_0_ = −37 mL/mol and *p*_0_ = 6800 bar. Red line: statistical-mechanical multistate cooperative model with volume change per amino acid residue Δ*v* = −2.0 mL/mol, *ν* = 129, *p*_0_ = 6800 bar. Cooperativity parameter σ = 5 × 10^–5^. (B) Free energy predictions of the three models. Blue line: chemical equilibrium two-state model (eq 4). Magenta line: statistical–mechanical two-state model (eq 9). Red line: multistate cooperative model (eq 13). Same parameters as in panel A.

Figure 1 shows a comparison of the three unfolding models. The parameters are chosen to provide the best fit to lysozyme pressure-unfolding as described by Figure 3 in ref (26). All three models provide an excellent fit to the experimentally observed sigmoidal unfolding transition Θ_u_(*p*). Moreover, the chemical equilibrium and the statistical-mechanical two-state model use the same set of parameters.

However, as discussed, the pressure profiles of the free energies are quite different. The chemical equilibrium model predicts a linear dependence with a positive free energy of 6.0 kcal/mol at 1 bar (blue line in Figure 1B). The statistical–mechanical two-state model predicts a zero free energy for the native protein which becomes negative already at the midpoint of unfolding (*F*(*p*_0_) = −RT_0_ ln 2) (magenta line in Figure 1B). At *p* > *p*_0_, the free energies of the two two-state models become identical. An again different result is obtained with the multistate cooperative model. The free energy of the native protein is also zero. However, the free energy of the denatured protein is distinctly more negative than the free energies of the other two models (red line in Figure 1B). At *p* = *p*_0_, the free energy is –2 kcal/mol at 25 °C. At 90% unfolding (*p* = 9100 bar) the predicted free energy of the multistate model is −12.5 kcal/mol.

### Multistate Cooperative Approximation

The above formalism can be approximated by a simpler expression, which avoids the matrix formalism. The partition function is approximated by the largest eigenvalue λ_0_ of the above matrix.^27^ This leads to the following partition function
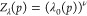
14with
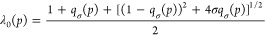
15The fraction of unfolded protein is given by
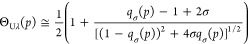
16The free energy of unfolding is
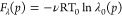
17For pressures *p* ≫ *p*_0_, the following approximation is valid.
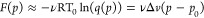
18Good agreement between the approximation (eq 16) and the matrix approach (eq 12) was obtained in the analysis of experimental data obtained for lysozyme,^26,28^ SNase^25^ and ribonuclease (RNase) A.^25^

## Results

We apply the multistate cooperative model for thermal, chemical and pressure denaturation to the essentially linear α-helical apolipoprotein A-1 (ApoA1) and the compact globular lysozyme. Despite their different molecular weights (see below) the two proteins are comparable because the native-to-unfolded transitions involve 120–130 amino acid residues in both cases. The multistate cooperative model for high-pressure unfolding is closely related to analogous models for chemical^22^ and thermal^18^ unfolding.

### Apolipoprotein A-1

Human ApoA1 is an amphiphilic protein of 243 amino acid residues (28.16 kDa) that binds to lipid membranes and is an essential constituent in the formation of high density lipoprotein (HDL) particles that are required in the reverse cholesterol transport. The crystal structure of C-terminal truncated ApoA1 (183 amino acids) is almost 100% α-helical.^29^ ApoA1 in solution has an α-helix content of 50–60% (∼125 amino acid residues).^13,14^ The unfolding of ApoA1 is thus a cooperative sequential α-helix ⇄ random coil equilibrium, which can be described conveniently with the statistical-mechanical multistate theory.^13,14,16^

### Lysozyme

Lysozyme (14.3 kDa) a 129-residue protein with ∼25% α-helix, ∼ 40% β-structure and ∼35% random coil in solution at room temperature.^15^ Upon thermal unfolding, the α-helix is almost completely lost and the random coil content increases to ∼60%.

The cooperative multistate theory is applied here to high-pressure unfolding and the results will be compared to thermal^15^ and chemical^22^ unfolding, also described previously with the cooperative multistate theory.^18,22^

### Pressure Unfolding of Apolipoprotein A-1 and Lysozyme

**Figure 2:**
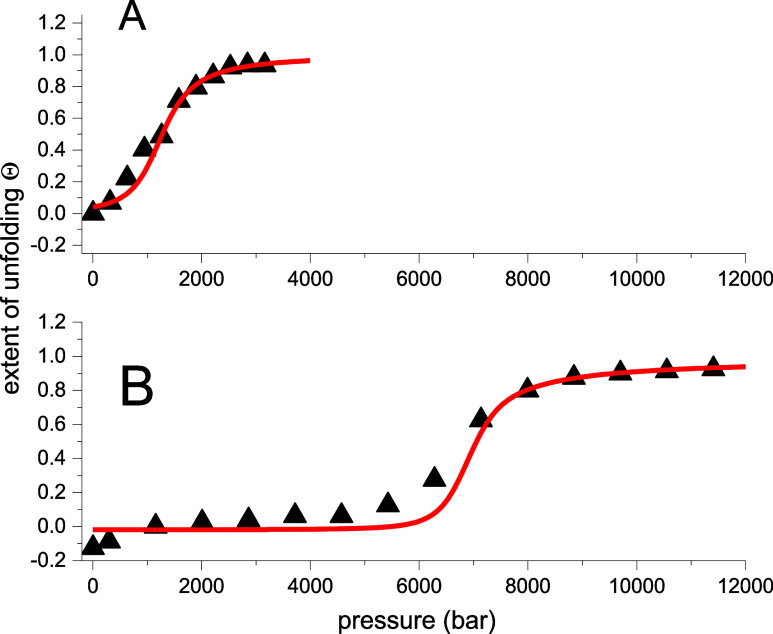
Pressure unfolding of ApoA1 and lysozyme at 25 °C. (A) Pressure unfolding of ApoA1 (black triangles). Experimental data obtained with FTIR-spectroscopy. Data taken from Mantulin and Pownall.^30^ Red line: multistate cooperative model (eq 12). Cooperativity parameter σ = 1.6 × 10^–3^, volume per amino acid residue Δ*v* = −3.8 mL/mol. (B) Lysozyme. Experimental data (black triangles) measured with FTIR-spectroscopy, data taken from Figure 3 of ref (26). Red line: multistate cooperative model (eq 12). Cooperativity parameter σ = 5 × 10^–5^. Volume change per amino acid residue Δ*v* = −2.0 mL/mol.

Figure 2 compares the pressure unfolding of apolipoprotein A-1 (ApoA1) with that of lysozyme according to eq 12.

The relevant parameters are summarized in Table 1.

**Table 1:** Pressure Denaturation of ApoA1 (Experimental Data from Mantulin and Pownall^30^) and Lysozyme (Experimental Data from Smeller et al.^26^)

parameters	units	ApoA1	lysozyme
number of amino acids, *N*_aa_		123	129
midpoint pressure, *p*_0_	bar	1150	6800
pressure at unfolding start, *p*_ini_	bar	1	5000
pressure at 90% denaturation, *p*_end_	bar	2501	9100
pressure diff. (*p*_end_ – *p*_ini_) Δ*p*	bar	2500	4100
cooperativity parameter, σ		1.6 × 10^–3^	5 × 10^–5^
volume change per aa residue, *dv*	mL/mol	–3.8	–2.0
volume change per protein, Δ*V*_0_	mL/mol	–467	–253
free energy change, Δ*F*	kcal/mol	–13.1	–10.4
Δ*p*Δ*V*_0_	kcal/mol	27.9	25.3
entropy change, Δ*S*	kcal/mol K	0.14	0.12

The open structure of ApoA1 with its linear α-helix is distinctly less stable than the more compact lysozyme. This is evidenced by the low midpoint pressure of 1150 bar. In contrast, the midpoint pressure of lysozyme is 6800 bar. The pressure range of unfolding is 2500 bar for ApoA1 but 4100 bar for lysozyme. Similar results for the pressure unfolding of lysozyme in H_2_O and D_2_O have been reported by Hédoux et al.^28^ Volume changes per amino acid residue are larger (−2.8 to −2.9 mL/mol) and the cooperativity parameters are σ = 5 × 10^–5^ and σ = 1 × 10^–4^, respectively.

**Figure 3:**
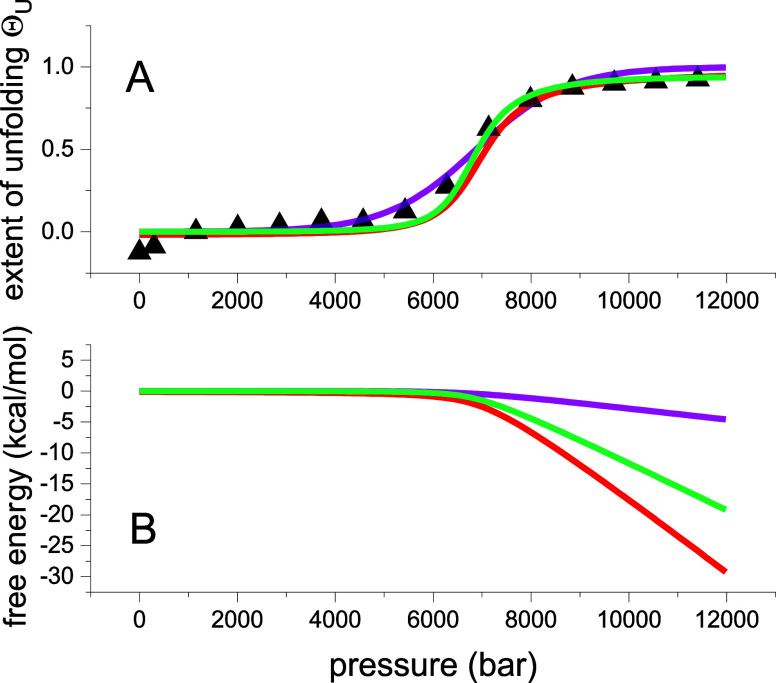
Pressure unfolding of lysozyme, modeled with 8 cooperative subdomains (green line), compared to the statistical-mechanical two-state model (magenta line) and the multistate cooperative model (with 129 aa) (red line). The parameters of the subdomain model are *ν* = 8 subdomains, Δ*V* = −20 mL/mol per subdomain and σ = 5 × 10^–2^.

Pressure unfolding of proteins is measured with spectroscopic methods that reflect volume changes only indirectly. The true volume reduction of the protein is not known. The multistate cooperative model offers a large range of different parameters to simulate the same experimental data (Δ*v*, ν and σ). This is demonstrated in Figure 3. The experimental data in Figure 3A are identical to those in Figures 1A and 2B and present the pressure unfolding of lysozyme. The green lines in Figure 3 represent a subdomain unfolding model. As an upper limit, we assumed eight subdomains for lysozyme, which under pressure move closer together with a volume reduction of −20 mL/mol per subdomain. The cooperativity parameter is σ = 5 × 10^–2^. The latter increases with the number of subdomains, and reflects a decrease in cooperativity. Figure 3B demonstrates that the free energy of this subdomain model is between those of the two-state model and the multistate cooperative model with ν = 129 amino acid residues with a volume reduction of −3 mL/mol per amino acid residue.

As a general conclusion it thus follows that spectroscopic measurements of pressure unfolding are not specific enough to allow an unambiguous interpretation of the unfolding process. A direct measurement of the volume reduction would indeed be necessary.

### Heat Denaturation of ApoA1 and Lysozyme

Proteins are denatured by very low or very high temperatures. Depending on the protein, the denaturation temperature varies strongly. The method of choice to study thermal unfolding of proteins is DSC.^31,32^ The heat capacity C_p_ is measured as a function of temperature and the important thermodynamic parameters of unfolding, that is, enthalpy Δ*H*(*T*), entropy Δ*S*(*T*) and free energy Δ*G*(*T*) are obtained directly by integration of the experimental *C*_p_(*T*) values.^11,18,19^ Spectroscopic measurements yield in contrast only indirect information on thermodynamic properties and can deviate substantially from DSC measurements.^15^

**Figure 4:**
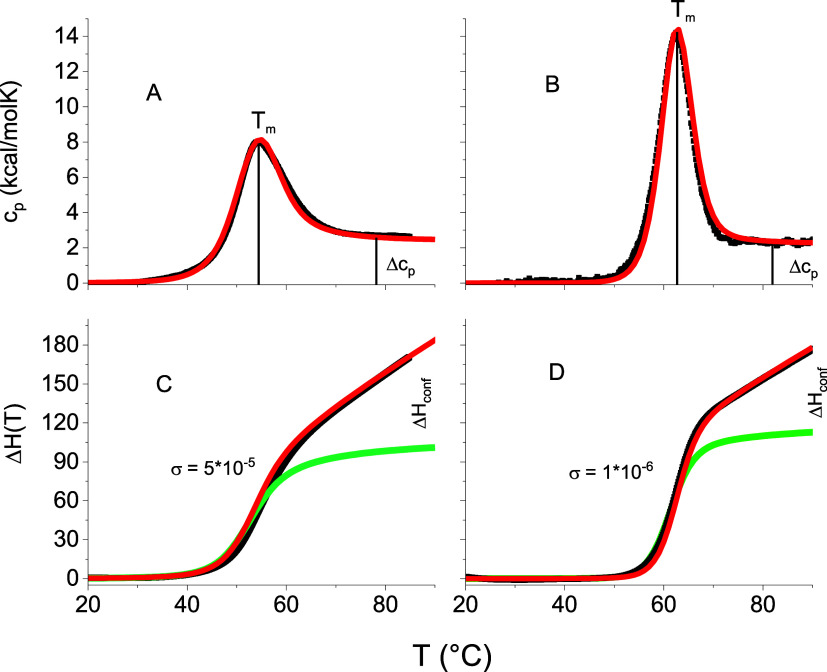
Heat denaturation of ApoA1 (A, C) and lysozyme (B, D) as measured by DSC. Black lines: about 2500 experimental DSC data points. Red lines: multistate cooperative model.^11,18,19^ Green lines: predicted enthalpy change Δ*H*_conf_ (*T*) of the conformational transition (A, B) Heat capacity *C*_p_(*T*). Midpoint temperatures *T*_0_ = 54 °C for ApoA1 (pH 7.4) and *T*_0_ = 63 °C for lysozyme (pH 2.5). (C, D). Unfolding enthalpies *H*(*T*) of ApoA1 and lysozyme obtained by integration of the heat capacities. The fit parameters and relevant thermodynamic parameters are summarized in Table 2.

Figure 4 displays DSC measurements of ApoA1 and lysozyme.

The simulation of the experimental data follows references.^11,18,19^ The simulation parameters are, *h*_0_, the enthalpy per amino acid residue, *c*_v_, the heat capacity per amino acid, and σ, the cooperativity parameter. The simulation parameters and other relevant thermodynamic results are summarized in Table 2.

**Table 2:** Thermal Unfolding of ApoA1 (Experimental Data Taken from Eckhardt et al.^16^) and Lysozyme (Experimental Data Taken from Li-Blatter et al.^21^)

parameters	units	ApoA1	lysozyme
number of amino acids, *N*_aa_		123	129
midpoint temperaature, *T*_0_	°C	54	63
temp. at unfolding start, *T*_ini_	°C	33	52
temp. at 90% unfolding, *T*_end_	°C	77	77
temp. diff. (*p*_end_ – *p*_ini_) Δ*T*	°C	44	25
cooperativity parameter, σ		5 × 10^–5^	1 × 10^–6^
enthalpy per aa, *h*_0_	cal/mol	900	950
heat capacity per aa, *c*_v_	cal/mol K	8	7
molar heat capacity change, Δ*c*_p_	kcal/mol K	2.72	2.48
conf. enthaply change, Δ*H*_conf_	kcal/mol	96	107
conf. entropy change Δ*S*_conf_	kcal/mol K	0.29	0.318
enthalpy change at 90% unfolding,Δ*H*	kcal/mol	148	146
free energy change at 90% unfolding, ΔF	kcal/mol	–8.51	–5.7
entropy change at 90% unfolding, Δ*S*	kcal/mol K	0.446	0.434

Thermal unfolding further confirms the lower stability of ApoA1 compared to lysozyme. The midpoint temperature of ApoA1 is 54 °C, that of lysozyme 63 °C. As the unfolding enthalpies of the two proteins are almost equal and *T*_0_ = Δ*H*_0_/Δ*S*_0_, the temperature difference is caused by differences in the unfolding entropy.

The unfolding temperature *T*_0_ of lysozyme depends on pH and increases with increasing pH.^32^ The present data (Figure 4) refer to a PBS buffer at pH 2.5.^15^ Hédoux et al. studied lysozyme unfolding with DSC and Raman spectroscopy in water.^28,33^ T_0_ is shifted to 74 °C but the heat of unfolding is in broad agreement with the results given in Table 2.

### Chemical Unfolding of Apolipoprotein A-1 and Lysozyme

**Figure 5:**
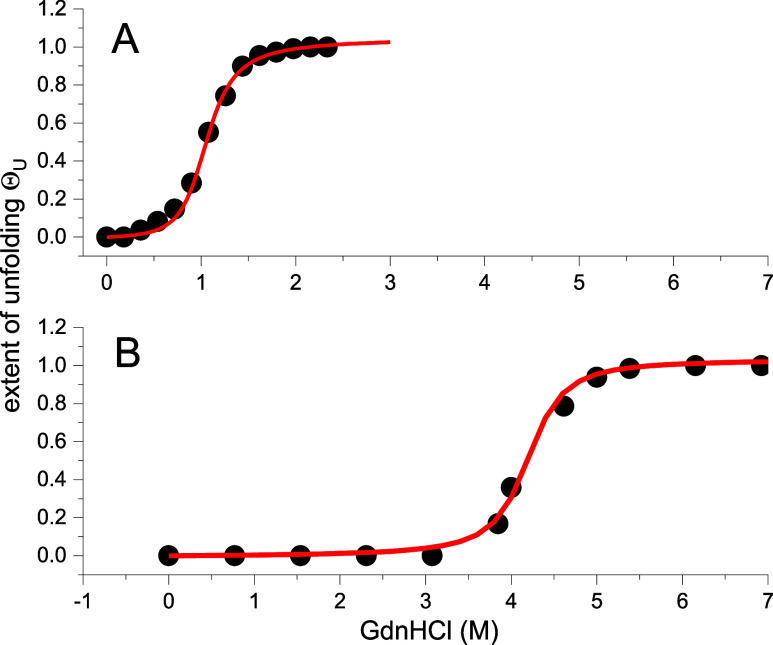
Chemical unfolding of ApoA1 and lysozyme. (A) ApoA1 (●). Experimental data.^30^ Midpoint concentration *c*_0_ = 1.07 M. Cooperativity parameter σ = 2 × 10^–2^. Guanidine HCl binding constant *K*_D_ = 0.95 M^–1^. (B) Lysozyme. (●) Experimental data.^34^ Midpoint concentration *c*_0_ = 4.2 M. Cooperativity parameter σ = 2 × 10^–3^. Guanidine HCl binding constant *K*_D_ = 0.245 M^–1^.

ApoA1 and lysozyme were denatured by adding increasing concentrations of guanidine HCl (GdnHCl). Figure 5 shows the sigmoidal transition curves for chemical unfolding. Mantulin and Pownall measured the chemical denaturation of ApoA1 in parallel to pressure unfolding.^30^ Lysozyme chemical unfolding was reported by Ahmad and Bigelow.^34^

All data are summarized in Table 3.

**Table 3:** Chemical Unfolding of ApoA1 (Experimental Data Taken from Mantulin et al.^30^) and Lysozyme (Experimental Data Taken from Smeller et al.^26^)

parameters	units	ApoA1	lysozyme
number of amino acids, *N*_aa_		123	129
*C*_gdm_ at midpoint of unfolding, *c*_0_	M	1.07	4.1
*c*_Gdm_ at start of unfolding, *c*_ini_	M	0.3	3.0
*c*_Gdm_ at 90% unfolding, *c*_end_	M	1.6	5.0
conc. range of unfolding, Δ*c*_Gdm_	M	1.3	2.0
cooperativity parameter, σ		2 × 10^–2^	2 × 10^–3^
binding constant of Gdm, *K*_D_	M^–1^	0.95	0.245
free energy change, Δ*F*, at 90% unfolding	kcal/mol	–30.9	–14.1
entropy change, Δ*S*	kcal/mol K	nd	0.28
enthalpy change, Δ*H*	kcal/mol	nd	70

The midpoint concentration for ApoA1 unfolding is only 1.07 M compared to 4.2 M for lysozyme. A low concentration of GdnHCl is sufficient to denature ApoA1. It is the result of the better binding of GdnHCl to ApoA1 (binding constant *K*_D_ = 0.9 M^–1^) than to lysozyme (*K*_D_ = 0.245 M^–1^). The cooperativity parameters are σ = 2 × 10^–2^ for ApoA1 and σ = 1 × 10^–3^ for lysozyme. The binding of the denaturant thus reduces the cooperativity in both proteins compared to pressure unfolding. Again, the unfolding of ApoA1 is less cooperative than that of lysozyme.

## Discussion

### Models for Pressure Unfolding of Proteins

Pressure unfolding of proteins has usually been described by a chemical equilibrium two-state model. Here, we propose a multistate cooperative model as a flexible and physically more realistic alternative. In the limit of no cooperativity this model degenerates into a statistical-mechanical two-state model (Figure 1, magenta line). Another limit of this model is a cooperative subdomain model (Figure 3, green line). A small number of protein subdomains interact cooperatively and move closer together to reduce the protein volume.

The multistate cooperative model for protein unfolding under pressure follows the same principles as discussed recently for thermal^11,18,19^ and chemical unfolding.^22^ It is thus possible to compare pressure denaturation with thermal and chemical unfolding. The three methods, pressure denaturation, heat unfolding and chemical unfolding lead to denatured proteins of different structure.^35^

### Pressure Unfolding versus Heat Unfolding and Chemical Unfolding

**Figure 6:**
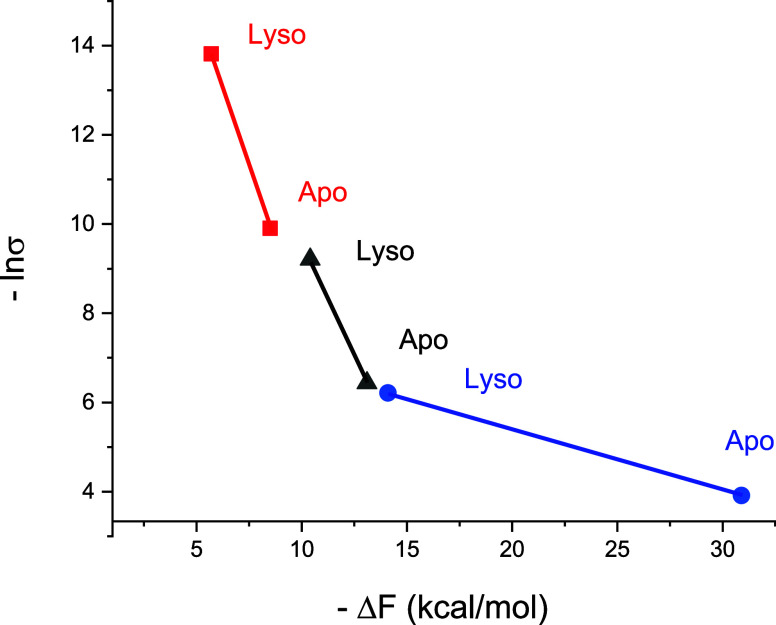
Comparison of the three unfolding processes using common parameters. The natural logarithm of the cooperativeity parameter, lnσ (proportional to the free energy of nucleation) is plotted vs the free energy released upon unfolding, Δ*F* for ApoA1 (Apo) and lysozyme (Lyso). Temperature-induced unfolding (red), pressure-induced unfolding (black) chemical unfolding (blue).

The common parameters of the different models are the free energy change involved in unfolding (Δ*F*(*p*) = −RT_0_ ln *Z*(*p*)) and the cooperativity parameter σ, where ln σ is proportional to the free energy of nucleation (Figure 6). These parameters are, on the one hand, characteristic of the nature of the proteins and on the other hand, yield information on the thermodynamics of the differernt unfolding processes.

Figure 6 shows the free energy of nucleation that is proportional to ln σ as a function of the free energy released upon unfolding Δ*F*. An approximately linear dependence is observed in the case of temperature and pressure denaturation.

When comparing the three unfolding methods (Figure 6), thermal unfolding is the most cooperative process (smallest σ), followed by decreasing cooperativity for pressure unfolding and chemical unfolding. ApoA1 displays a particularly low cooperativity in chemical unfolding. This can be explained by the strong binding of GdnHCl to ApoA1. GdnHCl binds to most proteins with a binding constant of *K*_D_ ∼ 0.25 M^–1^.^22^ The binding constant to ApoA1 is much stronger, *K*_D_ ∼ 0.95 M^–1^. The strong binding opens the protein structure and reduces the cooperativity.

Heat unfolding yields the smallest negative free energy change, followed by pressure denaturation, and chemical unfolding. An approximately linear relationship between the free energy change and lnσ (Figure 6) is observed for thermal and pressure unfolding. Lysozyme is generally slightly more cooperative and yields a less negative free energy change than ApoA1.

Interesting molecular insights are provided by considering the entropy change. The largest entropy change is observed for thermal unfolding with Δ*S* ∼ 0.44 kcal/mol for both proteins (Table 1). Chemical unfolding of lysozyme results in a lower entropy change of Δ*S* ∼ 0.28 kcal/mol,^22^ (Table 3) because part of the entropy gained upon unfolding is lost again upon binding of denaturants. The smallest entropy change is produced by pressure unfolding with Δ*S* ∼ 0.14 kcal/mol for ApoA1 and ∼0.09 kcal/mol for lysozyme (Table 2). This suggests that the structural changes induced by high-pressure are much smaller than those obtained with thermal and chemical unfolding.

### Volume Changes upon Pressure Application

The volume change upon unfolding, arises from a combination of several factors.^35^ A decrease in volume is caused by (i) the elimination of cavities and internal voids, (ii) the disruption of electrostatic interactions due to the electrostriction of water molecules around the unpaired charged residues and (iii) the hydration of charged and polar groups. The volume changes associated with the exposure of hydrophobic groups depend on the model compounds selected and fall into the range from small negative to positive values”. “These effects largely compensate for the increase in volume as the crystalline-like state of the protein interior is disrupted and exposed to solvent upon unfolding”. As demonstrated by high-precision densitometry, lysozyme revealed a small heat-induced volume increase and a GdnHCl induced volume decrease.^35^

Pressure denaturation of proteins generally leads to a decrease in volume, which is due to molecular compaction as illustrated with lysozyme.^36^ Unfortunately, this volume reduction has not yet been measured directly, but follows from the interpretation of spectroscopic unfolding measurements with unfolding models. For lysozyme the two-state models predict a volume reduction of −37 mL/mol, the subdomain model 8 × −20 = −160 mL/mol, and the multistate cooperative model 129 × −2 = −258 mL/mol. All three models describe the sigmoidal unfolding transition perfectly well.

We have analyzed pressure isotherms of ApoA1,^30^ lysozyme,^26,28^ SNase^25^ and RNase^25^ with the two-state models and the multistate cooperative model. The average volume change of six pressure isotherms of these proteins in the two-state analysis was −60 ± 16 mL/mol. For the multistate cooperative model, the average volume change per amino acid residue was −2.9 ± 0.7 mL/mol, which results in −350 ± 80 mL/mol as the average volume change of the whole proteins.

As there are no direct volume measurements, we estimate volume changes from the experimentally available protein compressibility. The protein compressibility  was measured as κ = 2 × 10^–11^ – 8 × 10^–11^ Pa^–1^ for SNase.^24^ The predicted volume change is thus Δ*V*(*p*) = −*V*_0_(1 – *e*^–κ*p*^). For a volume of *V*_0_ ∼ 10 L/mol (e.g., SNase) at a pressure of 3000 bar the predicted volume change, calculated with the compressibility κ = 8 × 10^–11^ Pa^–1^ is Δ*V* = −259 mL/mol. This calculation, while only approximate, shows that large volume changes can be expected and are consistent with the multistate cooperative model.

A molecular interpretation of volume changes under pressure has been given.^37^ Pressure reduces the void volume, but enhances the hydration sphere of the protein (see Figure 8) in ref (37). For a protein with 100–200 amino acid residues a volume change of∼ −100 mL/mol is predicted.^37^

**Figure 7:**
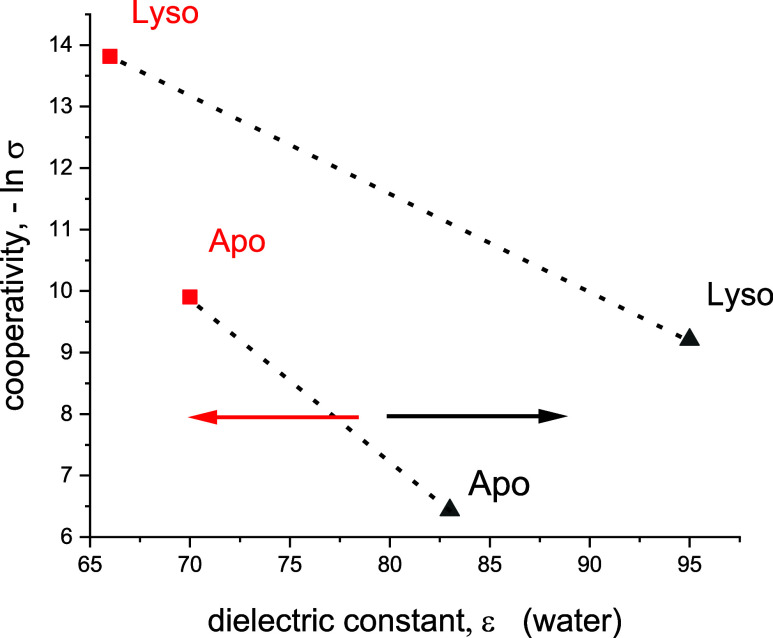
Cooperativity of unfolding (−ln σ) as a function of the dielectric constant, ε of water at the specific temperatures *T*_0_ (red) and pressures p_0_ (black) for ApoA1 and lysozyme. At room temperature and atmospheric pressure the dielectric constant of water is ε ∼ 80. Increasing the temperature reduces the dielectric constant of water (red arrow). Increasing the pressure enhances the dielectric constant of water (black arrow).

The different protein behaviors under high temperature and high pressure result to some extent from changes of the dielectric constant, ε, of water under the different conditions. An increase in temperature reduces the dielectric constant of water, whereas an increase in pressure enhances the dielectric constant of water. Figure 7 shows the cooperativity of unfolding as a function of the dielectric constant of water at the midpoint of temperature and pressure unfolding. Increasing the temperatue reduces the polarity of water, which facilitates water penetration into the more hydrophobic protein interior. Increasing pressure enhances the polarity of water. This reinforces hydrogen bonding between water molecules and reduces their penetration into the protein interior, leading to more pronounced hydration shells of the protein.

## Conclusions

Protein folding/unfolding is a cooperative process. Here, we propose a truly cooperative model for the high-pressure unfolding of proteins. The model involves the cooperative interaction of all amino acids of a particular protein, and can also handle the cooperative volume reduction of a small number of subdomains. In the limit of no cooperativity the model degenerates into a statistical-mechanical two-state model. The latter model is quite similar to the well-known chemical equilibrium two-state model, but predicts a different temperature profile of the free energy. In the multistate cooperative model the native protein is the reference state with free energy zero.

The new model is built on the same principles as described for the cooperative unfolding induced by heat or chemical agents. It is thus possible for the first time to draw a comparison between the three methods of protein denaturation. Thermal unfolding is the most cooperative process, followed by pressure unfolding and chemical unfolding. On the other hand, the change in free energy is less negative for thermal unfolding than for pressure unfolding and chemical unfolding. Particularly interesting is a comparison of the entropies. Pressure unfolding displays the smallest enthalpy change, which is probably indicative of the much smaller structural changes induced by pressure compared to heat and chemical agents. Examples are given for an essentially helical protein (ApoA1) and a globular protein (lysozyme). Although the differences between these two proteins are small, the data provide insight into the specific unfolding processes.
